# Rapidly lowering triglyceride levels by plasma exchange in acute pancreatitis: What's the point?

**DOI:** 10.1002/jca.21972

**Published:** 2022-02-23

**Authors:** Amanda J Berberich, Robert A. Hegele

**Affiliations:** ^1^ Department of Medicine Schulich School of Medicine and Dentistry, Western University London Ontario Canada; ^2^ Robarts Research Institute Schulich School of Medicine and Dentistry, Western University London Ontario Canada

Pancreatitis can be triggered by excessive quantities of large triglyceride (TG)‐rich chylomicrons in the serum, which begin to accumulate when serum TG levels exceed ~1000 mg/dL (or ~10 mmol/L).^1^ The risk of pancreatitis due to hypertriglyceridemia (HTG) increases as TG levels rise, but HTG is rarely a cause in isolation unless TG levels exceed ~2000 mg/dL (or ~20 mmol/L).^2^ The pathophysiology underlying HTG‐associated pancreatitis (HLAP) is incompletely understood. Some literature suggests that higher TG levels on presentation are associated with a less favorable prognosis.^3^ Once HLAP is triggered, it is also unclear if the persistent presence of chylomicrons perpetuates or prolongs the pancreatitis episode, or if rapidly lowering TG levels promotes more rapid recovery or improves clinical outcomes.

The TG contained within lipoproteins is hydrolyzed by lipoprotein lipase (LPL), an endothelial bound enzyme that cleaves TG into its component fatty acids and glycerol, resulting in removal of TG from the circulation.^4^ Dietary‐derived chylomicron particles are normally cleared within 3 to 4 hours of eating, but they accumulate abnormally when the processing capacity of LPL becomes overwhelmed. LPL has several cofactors, activators, and repressors that modulate its processing efficiency, although it retains at least partial function in almost all individuals who present with HLAP.^4^ The exception would be individuals with biallelic pathogenic variants in the *LPL* gene itself or genes for key cofactors causing familial chylomicronemia syndrome: a rare condition affecting ~1 in 300 000 to 1 million people.^5^ When an ongoing dietary source of TG is no longer available, by withholding oral intake (ie, nil per os or NPO), even compromised LPL will steadily process the accumulated chylomicron burden, leading to a predictable and fairly rapid exponential fall in serum TG levels.^6^, ^7^

For the inexperienced clinician, the presentation of acute pancreatitis in the context of lipemic opalescent plasma in a patient with chylomicronemia can be dramatic and compelling. It can elicit an understandable desire to eradicate or normalize the metabolic disturbance as quickly as possible. Despite lack of evidence for benefit,^7^ therapeutic plasma exchange (TPE) has been suggested as a method to rapidly lower TG levels in the setting of HLAP,^8^ although prospective head‐to‐head trials comparing clinical outcomes against conservative methods are conspicuously absent. Additionally, TPE is costly, requires specialized staff and equipment, and is not without risk. Therefore, a potential role for TPE in the management of HLAP remains an unresolved, but important clinical question.

An original article in the *Journal of Clinical Apheresis* by Chen et al^9^ helps to answer questions from the clinician who wonders about the benefit of one (n = 29) or two (n = 17) courses of TPE for acute pancreatitis due to chylomicronemia. Chen et al report the biochemical and clinical trajectory of an observational cohort of 181 individuals with HLAP managed either conservatively (n = 135) or with TPE (n = 46). Their study was conducted at a single center in China. The two groups were roughly similar for most clinical and biochemical features at baseline, with some modest differences in a few variables suggesting that those who received TPE were somewhat sicker.

The main outcomes were plasma TG levels, specifically the proportion of patients attaining TG < 500 mg/dL within 48 hours. Using this rather arbitrary metric, the authors report a higher odds ratio (OR) that the TPE group attained this level compared with the non‐TPE group at 24 hours (OR 2.74; 95% confidence interval 1.30‐5.79, *P* = .008), among those with measured values (n = 97 for conservatively managed individuals and n = 44 for TPE patients). This difference persisted, although attenuated at 48 hours and completely disappeared by 72 hours. Considering only the mean absolute TG reductions at 24, 48, and 72 hours for the TPE and non‐TPE groups, there were, respectively, 70.9% and 48.2% (*P* < .001), 76.4% and 70.9% (*P* = .023) and 77% in both groups (NS). There were no differences in 28‐ and 90‐day mortality rates, although rates of acute respiratory distress syndrome (ARDS) and severe kidney injury were significantly higher in the TPE vs non‐TPE group. This indicates either that patients who received TPE were sicker at the outset or perhaps there was a deleterious unintended outcome related to TPE. Additionally, those receiving TPE had a longer (12.5 vs 8.0 days; *P* < .001) and more expensive (41.04 vs 12.16 thousand Yuan; *P* < .001) hospital stay compared with those managed conservatively.

The authors are to be congratulated for assembling these data, which are useful for clinicians who encounter this scenario. Although it is an observational and non‐randomized study, it provides a rare head‐to‐head assessment of the change in TG levels in patients with HTG‐associated pancreatitis treated with TPE or conservatively without TPE. The findings also permit evaluation of the incremental benefit of TG reduction afforded by TPE over conservative measures such as NPO, fluids, pain control and insulin when necessary. The findings indicate that by 24 hours after intervention, TG falls by ~50% with conservative measures alone and that TPE provides an additional ~20% reduction at 24 hours that is largely lost after 48 and is absent at 72 hours. There is no difference in clinical outcomes acutely, although in the long term there was a higher incidence of ARDS and renal injury in the TPE group, suggesting that these patients were sicker at the outset, which may have been one reason why TPE was pursued.

Given the reduction of ~50% in the conservative group compared with ~70% in the TPE group at 24 hours, it is clear that most of the heavy lifting for TG reduction is accomplished by conservative measures. The real question is whether the 20% extra TG reduction from TPE confers any actual clinical “benefit”. Clearly, TPE bends the TG curve acutely, but after 48 and 72 hours conservative measures largely catch up objectively, with no obvious clinical implications. These observations in one of the largest cohorts yet examined suggest that there is no short‐term or long‐term benefit to TPE in this situation. The acute pancreatitis event has already occurred, and TPE neither facilitates resolution of the incipient episode nor prevents another acute event within a 72 hour time frame. The outstanding questions related to possible clinical benefit of TPE in HLAP would be resolved by a randomized clinical trial.

The observations from Chen et al are a valuable addition to the literature and indicate that there is only transient incremental benefit to TG lowering with TPE vs conservative management that is largely gone by 48 hours, and disappears fully by 72 hours, with no acute benefit seen for clinical end points. Thus, TPE in the acute situation does not seem to accomplish anything more than conservative measures. Chronically, there is no role for TPE in ongoing management or prevention, since rebound of severe HTG always occurs unless the primary cause of the HTG is controlled medically, first by eliminating the precipitating factors and second by using targeted medical management of HTG with diet, weight loss, diabetes management, fibrates, eicosapentaenoic acid and new biologics including anti‐APOC3 and anti‐ANGPTL3 agents.^10^, ^11^ TPE seems to afford no long‐term benefit either in terms of mortality or long‐term complication rates. A randomized clinical trial would help settle questions about the value of TPE in HALP once and for all.

## CONFLICT OF INTEREST

Amanda J Berberich has no conflicts to report. Robert A. Hegele has received honoraria from Akcea‐Ionis, Amgen, Arrowhead, HLS Therapeutics, Novartis, and Pfizer.
